# Comparison of the effectiveness of instrument-assisted soft tissue mobilization and extracorporeal shock wave therapy in myofascial pain syndrome

**DOI:** 10.55730/1300-0144.5753

**Published:** 2023-10-16

**Authors:** Şeyda CANDENİZ, Seyit ÇITAKER, Gökhan MARAŞ, Hatice Esra YAVUZER, Hasan YILDIRIM, Zafer GÜNENDİ

**Affiliations:** 1 Department of Therapy and Rehabilitation, Kızılcahamam Vocational School of Health Services Ankara University, Ankara, Turkiye; 2 Department of Physiotherapy and Rehabilitation, Faculty of Health Sciences, Gazi University, Ankara, Turkiye; 3 Department of Physical Medicine and Rehabilitation, Kızılcahamam Public Hospital, Ankara, Turkiye; 4 Faculty of Kamil Özdağ Science, Department of Mathematics, Karamanoğlu Mehmetbey University, Karaman, Turkiye; 5 Department of Physical Medicine and Rehabilitation, Faculty of Medicine, Gazi University, Ankara, Turkiye

**Keywords:** Myofascial pain syndrome, instrument-assisted soft tissue mobilization, extracorporeal shock wave therapy, pain, range of motion, pressure pain threshold

## Abstract

**Background/aim:**

To compare the effectiveness of instrument-assisted soft tissue mobilization (IASTM) and extracorporeal shock wave therapy (ESWT) used in myofascial pain syndrome (MPS) and to determine whether they are superior to conservative treatment (CT).

**Materials and methods:**

A total of 42 female patients (aged 18–60 years) diagnosed with MPS were enrolled and randomly assigned to either the CT (n = 14), CT+IASTM (n = 14), or CT+ESWT group (n = 14). All of the groups received treatment for 3 weeks (CT: 5 sessions per week, 15 sessions in total, ESWT and IASTM: 2 sessions per week, 6 sessions in total). Neck stretching exercises were given to all of the patients as a home program. The pain intensity of the patients was determined using the visual analog scale (VAS). The pressure pain threshold (PPT) was measured with an algometer. Cervical joint range of motion (ROM) was measured with a cervical ROM (CROM) device. Pain, cervical disability, quality of life, and sleep disturbances were evaluated with the Neck Outcome Score (NOOS). Depression and anxiety parameters were evaluated with the Hospital Anxiety and Depression Scale (HADS). Evaluations were made before treatment and 3 days after the last treatment session.

**Results:**

The CT+IASTM group was more successful than the other groups in terms of pain intensity, PPT, and improvements in the ROM parameters (p < 0.05). No significant difference was found between the NOOS and HADS scores of the groups when the posttreatment changes were compared to pretreatment (p > 0.05).

**Conclusions:**

All 3 of these treatments can be used to alleviate the negative effects of MPS. IASTM treatment can be preferred primarily in the creation of combined treatment programs for patients with ROM limitations and low PPTs.

## 1. Introduction

Myofascial pain syndrome (MPS) is a common extraarticular musculoskeletal disorder characterized by myofascial trigger points. The prevalence of MPS in patients admitted to medical clinics because of pain ranged from 30% to 93% [1]. This syndrome manifests with clinical pain, limited range of motion (ROM) in the joints, and myofascial trigger points. The purpose of the treatment of MPS is to inactivate the trigger points, reduce the tension and adhesions in the tissue, and eliminate chronic pain and complaints [2].

Many invasive and noninvasive treatments are used to treat MPS. Superficial heat agents, transcutaneous electrical nerve stimulation (TENS), ultrasound (US), laser, trigger point injection, extracorporeal shock wave therapy (ESWT), exercise, spray and stretching, balneotherapy, massage, soft tissue mobilization, ischemic compression, dry needle, acupuncture applications, and pharmacological agents are among these treatments [3–9]. Despite a wide and different treatment spectrum, clinical evidence for optimal treatment is reported to be insufficient [10].

In studies on MPS, conservative treatment (CT) protocols consisting of hotpack (HP), TENS, US interventions, and neck stretching exercises are all frequently used and combined with other treatments [10–15]. The success of TENS in reducing pain intensity and trigger point sensitivity in the clinical management of MPS has been demonstrated by extensive qualitative and quantitative studies [16]. Studies have validated the central analgesic mechanism, which is that TENS treatment for MPS significantly reduces substance P overexpression, increases opiate receptor expression in the parabrachial nucleus, and boosts c-Fos expression in the rostral ventromedial medulla [17,18]. Similarly, there are numerous clinical studies that support the usefulness of US in effectively reducing MPS symptoms [10–13]. By enhancing blood vessel and cell membrane permeability and fostering angiogenesis and microcirculation, US can reduce MPS pain by encouraging muscle relaxation and extending connective tissue [11].

The use of ESWT in the treatment of MPS has been increasing in recent years. The treatment is based on the application of high-pressure sound waves to the target tissue through an applicator. There are various hypotheses about the mechanism of action of ESWT. It was reported that shock waves increase blood supply through angiogenesis in ischemic muscle, modulate the functions of ion channels, resorb calcific deposits, alleviate pain, and produce substance P [19]. Studies have shown that ESWT reduces pain and disability while increasing ROM in MPS [20–22].

Another method used in the treatment of MPS is instrument-assisted soft tissue mobilization (IASTM), which is a mobilization method applied using steel instruments designed to reduce pain and limitations and increase soft tissue mobility [23]. It stimulates the remodeling process by creating microtrauma on the soft tissue, aiding in the resorption of fibrosis and the healthy and functional alignment of new collagen deposition [24]. In studies conducted on patients with MPS, it was reported that IASTM increases the pressure pain threshold (PPT), provides an increase in ROM, and significantly reduces pain and disability [25–27].

While extensive research has been done regarding the treatment of MPS and studies have shown some degree of efficacy, there is still a gap in research regarding the most effective treatment strategies being used. Therefore, the rationale of this study was to fill this gap, compare the effects of IASTM and ESWT used in the treatment of MPS, and determine whether they are superior to CT.

## 2. Materials and methods

### 2.1. Study design

This study was a 3-arm, parallel, nonblinded randomized clinical trial designed according to the Consolidated Standards of Reporting Trials guidelines [28] The study began after reveiving approval from the Gazi University Faculty of Medicine Clinical Research Ethics Committee (decision number 427-2022). The investigation was conducted in conformity with the guidelines of the Declaration of Helsinki and Good Clinical Practice. All of the participants signed a written informed consent form before the study began.

Female patients, aged 18–60 years, who presented to the Gazi University Faculty of Medicine, Department of Physical Medicine and Rehabilitation, with complaints of pain in the neck and upper back region, were diagnosed with MPS by a specialist physician, and met the inclusion criteria were included in the study. Patients over the age of 18, diagnosed with MPS according to Simon’s Diagnostic Criteria, had a trigger point in the trapezius muscle, and had not received any treatment for MPS in the last month were included in the study. The exclusion criteria were as follows: severe cervical disc disease, allergic skin disease, acute rheumatic disease, tumoral disease, fibromyalgia diagnosis, mental or psychotic disorders, venous insufficiency, presence of active infection, and pregnancy. In addition, it was planned to exclude patients from the study if they exhibited any contraindications for IASTM or ESWT [29,30]. Detailed anamnesis of the patients who were included in the study was taken, and detailed examinations were made by a specialist physician. Moreover, requests were made for the laboratory and imaging methods necessary for the exclusion of other diseases. Patients who met the inclusion criteria were randomized into 3 equal groups using the sealed envelope method. Intervention cards, including random assignment, were prepared by a researcher who was not involved in the clinical research and placed in sealed opaque envelopes. When a patient consented to participate in the trial, an envelope was drawn, and the patient was presented with the designated treatment regimen.

### 2.2. Outcome measures

The age, height, weight, body mass index (BMI), marital status, occupation, cigarette-alcohol use, presence of comorbidities, drugs used, dominant hand side, and localization of pain of the patients were asked about and recorded.

#### 2.2.1. PPT

A digital algometer device (Lafayette Instruments, Lafayette, Indiana) was used for the PPT evaluations. The PPT was measured from the most painful trigger point. The tip of the algometer was placed at a 90° angle to the area with the greatest sensitivity, and the pressure was increased until the patient experienced pain. The PPT was established as the pressure value at which the patient expressed pain. The average of the values obtained by taking the measurement 3 times was recorded in kilograms per square centimetre [31].

#### 2.2.2. Cervical joint ROM

Degrees of flexion, extension, right rotation, left rotation, right lateral flexion, left lateral flexion, cervical joint ROM obtained during active joint movement of the cervical region were measured with a cervical ROM (CROM) device (Performance Attainment Associates, St Paul, MN, USA) [32]. Each movement was measured 3 times and the average of the data was recorded on the evaluation form.

#### 2.2.3. Visual analogue scale

The pain scores of the patients were evaluated with the visual analogue scale (VAS) [33]. The meaning of the line placed between 0 and 10 on a 10-cm scale was explained to the patients. The absence of pain was described as 0, and the most severe pain to be felt was 10. According to these explanations, the patients were asked to mark their pain at rest, during activity, and at night on a 10-cm line. The marked place was measured in centimeters and recorded.

#### 2.2.4. Neck Outcome Score questionnaire

Disability, cervical mobility, quality of life, and sleep parameters were evaluated with the Neck Outcome Score (NOOS). The NOOS is a questionnaire consisting of 5 subcategories that question neck mobility, symptoms, participation in activities, sleep, and quality of life. It consists of 34 questions in total. Each question gets a score between 0 and 4, and the total score is between 0 and 100. The lowest score of 0 indicates serious problems and/or functional limitations; 100 asymptomatic healthy individuals are considered. The Turkish version of the questionnaire was made, and it was found to be valid and reliable [34].

#### 2.2.5. Hospital Anxiety and Depression Scale (HADS)

Depression and anxiety parameters were determined using the HADS. The scale was adapted to Turkish by Aydemir et al. [35]. Each question is scored between 0 and 3 on the scale, which consists of 14 items in total. The lowest score that individuals can get from both subcategories is 0, and the highest score is 21, indicating the maximum risk level.

#### 2.2.6. Level of satisfaction

The level of satisfaction was evaluated by the feedback the participants gave about the success of the treatment they received. Their responses were categorized as worsening, no change, and improvement compared to pretreatment.

All of the evaluations and measurements were made before treatment and 3 days after the last treatment session.

### 2.3. Interventions

#### 2.3.1. CT Group

The first group received a CT program including HP, US, and TENS, which is routinely given in physical treatment clinics. The HP, US, and TENS applications performed within the scope of the CT were applied for 3 weeks, with 5 sessions per week, for a total of 15 sessions. The patients were treated with HP for 20 min, conventional TENS for 20 minutes, and 1.5 W/cm^2^, 1 MHz of continuous therapeutic US with the trigger point as the center for 5 min [36,37]. The CT protocol was applied to all of the groups in the same way. Moreover, a home exercise program consisting of neck stretching exercises was given to all of the participants. Stretching exercises were given for the neck flexors, posterior cervical and suboccipital muscles, levator scapula, upper trapezius, and neck lateral flexor muscles. The patients were asked to perform these exercises bilaterally for 3 weeks, with 10 repetitions twice a day [38].

#### 2.3.2. IASTM

For the IASTM application, the patients were seated on a chair and positioned forward with support. During the application, the lubricity of the tissue was increased by applying baby oil to the relevant area. IASTM instruments made of stainless steel were applied to the neck and upper back muscles on the aching side at only 30º and 60º angles with the sweep technique on the origo and insertio lines at 60 repetitions per min [29]. Practice sessions lasted an average of 5 min. A total of 6 sessions (2 sessions with a 3-day interval per week) were applied for 3 weeks.

#### 2.3.3. ESWT

The most painful trigger point was identified and marked by the examining physician. Interventions were made on these marked points. The treatment was administered at the dose specified in the guidelines published by the International Society for Medical Shock Wave Treatment [39]. US gel was used to ensure conductivity during the intervention. A total of 6 sessions were applied on the muscle with an active trigger point at 1.5–2.0 bar at a frequency of 10 Hz with 2000 beats per session twice a week. The latent trigger points of the patients were not taken into account. The treatment was administered by a research physiotherapist experienced in ESWT. At the end of the treatment, the application area was gently wiped with a sterile sponge. After the last treatment session, 3 days were allowed to pass for extinction, and all of the evaluations made before the treatment was applied again, 3 days after the last session. In addition, the patients did not use any other drugs or supplements during the treatment process.

### 2.4. Statistical analysis

The IBM SPSS, Minitab, and R programs were used to obtain the findings. The comparisons of the demographic variables were made in terms of the treatment types, and assumptions such as normality and homogeneity of variance required by each test for the quantitative variables were checked before analysis. Normality assumption was checked with the Shapiro–Wilk Test as well as skewness/kurtosis values and Z-scores. The homogeneity of variance was checked with the Levene test. The chi-squared test (Pearson’s chi square) was used for the categorical variables and the 1-way analysis of variance (ANOVA) was used to compare the demographic variables in the different treatment groups. Moreover, mixed measures analysis of variance (also known as 2-factor mixed ANOVA) was applied to compare the repeated measures before and after treatment in the treatment groups and examine their interactions. Statistical significance was accepted as 5%. The margin of error was fixed at 5% by applying Bonferroni correction in multiple comparisons of significant statistical results.

### 2.5. Sample size and power of the study

The overall sample size required to achieve an adequate power of 0.95 was determined to be 39 individuals during the prestudy power analysis process using the partial eta squared (*η*^2^) value, taken as 0.10 (mean of the middle and upper reference ranges suggested by Cohen corresponds to the effect size = 0.33) at a significance level of 0.05 [40]. In consideration of potential sample losses, a total of 42 individuals, with 14 in each group, participated in the study. The power value that was determined after the study also supported that the sample used was enough.

In the poststudy power analysis process, the Cohen F effect size was calculated as 0.5403 and the average correlation coefficient between repeated measurements was calculated as 0.852 from the data obtained from a total of 42 individuals at a significance level of 0.05, and the power value was found to be 0.9936 with G*Power 3.1.9.7.

## 3. Results

The Figure demonstrates the study flow diagram. A total of 55 patients with MPS were assessed for eligibility. Of these, 10 patients did not meet the inclusion criteria, and 3 patients refused to participate in the study. The 42 patients (aged 18–60 years) enrolled in the study were randomized into 3 groups. As there was no withdrawal during the 3-week follow-up, 14 participants from each group completed the study. No significant adverse events were reported in any of the groups after the interventions.

Comparisons of the demographic variables were made in terms of the treatment types at the first stage, and the results of the comparison of the quantitative variables are given in Table 1. The results obtained for the qualitative variables are given in Table 2. Based on the descriptive statistics, the groups were close to each other in terms of the demographic and clinical measurements and were homogeneous in terms of these characteristics (p > 0.05).

When the pretreatment scores of the groups were compared, the VAS night pain intensity and right rotation ROM values in the CT+IASTM group were significantly lower than in the other groups (p < 0.05). The algometer values in the CT+IASTM group were significantly higher than in the other groups (p < 0.05). There was no difference between the groups in the pretreatment scores of the NOOS and HADS (p > 0.05) (Table 3).

Comparisons of the VAS scores within and the between groups before and after treatment are given in Table 4. In the within-group evaluations, significant decreases were detected in all of the groups in terms of the rest, activity, and night pain intensity after treatment when compared to the pretreatment status (p < 0.001). In the comparisons between the groups, no superiority was found in terms of the change in pain intensity at rest and at night (p > 0.05). On the other hand, CT+IASTM was significantly more effective than the other treatments in reducing the severity of activity pain (p < 0.05). CT+ESWT was also significantly more effective in reducing the severity of activity pain than CT (p < 0.05).

The ROM findings are given in Table 5. In the within-group evaluations, the ROM values in all directions in the CT+IASTM and CT+ESWT groups increased at significant levels after treatment when compared to their pretreatment status (p < 0.001). In the CT group, a significant increase was detected in the flexion, extension, and right and left lateral flexion movements after treatment (p < 0.001), but the change in the right and left rotation movements was not significant (p > 0.05). CT+ESWT was significantly more effective than CT alone in increasing neck extension and right-left lateral flexion ROM (p < 0.001). In the comparisons between the groups, CT+IASTM was significantly more effective than the other treatments in increasing cervical extension, right and left lateral flexion, and right and left rotation ROM (p < 0.001).

The findings of the changes in PPT are given in Table 6. The PPT values increased significantly after treatment in all of the groups when compared to the pretreatment values (p < 0.001). In the comparisons, CT+IASTM increased the PPT more than the other treatments (p < 0.001). CT+ESWT was significantly more effective than CT in increasing the PPT (p < 0.001).

Findings on the effects of the treatments on neck mobility, symptoms, sleep, activity and pain, quality of life, depression, and anxiety levels are given in Table 7. It was determined in the group evaluations that CT+IASTM and CT+ESWT contributed significantly to increasing mobility and quality of life and reducing disability, depression, and anxiety (p < 0.001). CT, on the other hand, provided a significant increase in mobility and quality of life, and a significant decrease in disability (p < 0.001), but did not have a significant effect on anxiety or depression (p > 0.05). In the evaluations between the groups, all 3 treatments had similar effects on the neck mobility, symptoms, sleep disturbance, activity and pain, quality of life, depression, and anxiety levels (p > 0.05).

The satisfaction of the patients with the treatment was significantly higher in all of the groups (p < 0.05). Although all of the patients in the CT+IASTM group reported improvement after treatment when compared to pretreatment, 85.7% of the patients in the CT+ESWT and CT groups reported improvement when compared to pretreatment. Regarding the success of the treatments in patient satisfaction, the percentage of satisfaction levels in the CT+IASTM group was statistically significantly higher than in the other groups (p < 0.05) (Table 8).

## 4. Discussion

The early efficacy of CT used in MPS rehabilitation and the ESWT and IASTM treatments applied in addition to this treatment were investigated in the present study. The effects of the treatments on pain intensity, PPT, and ROM, neck mobility, sleep disturbance, activity-related pain, quality of life, anxiety, and depression were evaluated. Clinically significant improvements were found in the mentioned parameters in all 3 groups, and CT+IASTM was more effective in terms of a decrease in pain intensity, an increase in PPT values, and an increase in ROM. CT+ESWT, on the other hand, was more effective in increasing PPT and ROM values when compared to CT. All 3 treatment modalities were equally effective in reducing symptoms, disability, anxiety, and depression, and improving sleep and quality of life.

The fact that the sociodemographic characteristics of the participants were similar provided homogeneity in the groups and prevented possible bias. It was reported in previous studies that MPS is most common in individuals aged 27–50 years [41]. In the present study, patients between the ages of 18–60 years old were included, and the mean age was between 27 and 36 years. In the study of Erden et al. [25], participants between the ages of 18 and 50 years were included, and the mean age was 36 years. Similarly, in a study conducted by Harris et al. [42], on individuals with MPS, the participants were between the ages of 18 and 40. The mean ages of individuals included in the studies were generally compatible with each other. The difference between the current study and other studies is that only female participants were included herein. Moreover, the fact that all of the patients were female eliminated the effects of biological and gender differences on treatment outcomes in terms of factors such as pain sensitivity, ligamentous laxity, hormonal cycles, and psychic predispositions.

According to the results obtained herein, all 3 treatments showed positive effects on the pain parameter of the MPS patients, and a statistically and clinically significant decrease was found in pain intensity in all of the groups when compared to the VAS values. Although no significant differences were detected between the groups in terms of decreases in pain intensity in night and rest parameters, the most significant decrease was in the CT+IASTM group for pain intensity in activity. The significantly increased ROM and PPT values in the IASTM group compared to the other groups may have contributed to the wider ROM during activities and less sensitivity in trigger points during muscle contractions, resulting in significant differences in the VAS activity values. As there is less requirement for joint ROM and contraction during rest and night than during movements, the discrepancy between the treatments may have become more obvious during activity. Considering that the pain seen in MPS increases with activity and its severity can change during the day, changes in pain caused by movement are very important [43]. The findings obtained herein also support this view. In addition, both CT+ESWT and CT+IASTM were more effective than CT in reducing pain during activity. IASTM loosens the fascial chain through mobilization and friction. Moreover, an increase is achieved in the tissue temperature and blood flow because of the friction created by the rhythmic strokes between the instrument and the tissue [44,45]. In this way, increasing tissue perfusion and contributing to the removal of local waste metabolites may explain the success of IASTM. Another effect of the application in pain management may be that the pressure created by the instrument on the tissue stimulates the A-beta sensory fibers more than in other treatments [46]. When the literature was reviewed, many studies were found in which all 3 treatments were frequently used in MPS rehabilitation. In the study of Ramadan et al. [14], in which they examined the effectiveness of IASTM applied in patients with myofascial trigger points, a significant improvement was reported in pain severity. This is also consistent with the findings of Motimath et al., who concluded that IASTM can reduce pain rapidly in the acute period [47].

According to the results obtained herein, although the groups did not have superiority over each other in terms of the amount of increase in neck flexion movement, more significant increases were found in all of the other ROMs in the CT+IASTM group. In IASTM, sweeping strokes are performed on the tissue, whereas in ESWT, controlled inflammation is induced on the trigger point through pressure pulsations. In IASTM, the implementation of these strokes, the resorption of calcific deposits, and allowing metabolites to enter the circulation more easily, and also the fact that ESWT application is painful, may have turned the results in favor of IASTM in the early period. It is possible to argue that rhythmic stroking interventions to the posterior of the neck and upper back with IASTM have a greater effect on reducing the stress on the tissue and relaxing the myofascia when compared to other interventions. In the pilot study of Erden et al. [25], in which they compared the efficacy of CT and IASTM in individuals with MPS, IASTM was found to be more effective in increasing ROM than CT in the short term. In the study of Harris et al. [42], individuals with MPS with a trigger point in the trapezius muscle were divided into 3 groups as the IASTM, sham IASTM, and control groups. They reported that IASTM was more effective on ROM compared to the control group in the immediate postintervention measure.

It was found that CT+ESWT was more effective than CT in increasing the neck right-left lateral flexion and extension ROM in the present study. For possible causes of significant improvements in lateral flexion and extension, it would be helpful to address the role of the upper fibers of the trapezius muscle. When the upper trapezius fibers contract unilaterally, they cause lateral flexion to the same side, and as a result of their bilateral contraction, they reveal the extension movement. In MPS, when a tense and shortened trapezius muscle tries to perform extension and lateral flexion movements, pain and tension will cause these movements to be limited. The focus of ESWT on the trigger points, which are the source of the pain, may have caused more significant improvements in ROM by reducing pain more effectively during these movements. Additionally, the CT+ESWT group in the current study showed significantly greater results in terms of pain intensity and trigger point sensitivity when compared to the CT group, which could be responsible for the increases in ROM.

Herein, a decrease in trigger point sensitivity was detected along with a statistically significant increase in PPT values in all of the groups. When the increases in the PPT values were examined in terms of effect sizes, the increases in both the CT+IASTM and CT+ESWT groups were significantly higher than those in the CT group. CT+IASTM also gave significantly better results than CT+ESWT. These results show parallelism with the decrease in pain intensity between the groups. Based on this outcome, it is concluded that the decrease in the pain intensity is proportional to the increase in the PPT values. In the study of Portillo-Soto et al. [48], the effects of massage and IASTM on trigger points were investigated. They reported that both interventions increased the tissue temperature and blood flow to the area. They emphasized that making effleurage-like, petrissage-like movements on the tissue with the aid of the instrument contributes to reducing sensitivity to pain by increasing blood flow to the region and removing local metabolites from the trigger point area. It was reported by Gulick et al. [26] that a 5-min intervention using 3 IASTM techniques effectively increased PPT values in 6 sessions over 3 weeks in individuals with MPS. In another study conducted by Shamseldeen et al. [49], it was stated that PPT values increased significantly compared to pretreatment after IASTM in individuals with MPS, 2 times a week, in a total of 4 sessions.

According to the results obtained herein, all 3 treatments were effective in increasing neck mobility. When the intergroup effectiveness of the treatments was examined, although the high scores obtained in the CT+IASTM group as a result of their answers to the questionnaires mean that the improvements were higher in this group, this increase was not statistically significant. The fact that individuals in the CT+IASTM group reported more improvement in cervical mobility was consistent with the result that CT+IASTM is more effective in terms of reductions in pain severity and increases in ROM when compared to other treatments.

When the severity of the symptoms associated with MPS and the success of the treatments in eliminating these symptoms were evaluated, the scores were increased in all 3 groups when compared to pretreatment. In this section, where headache, dizziness, and concentration impairment because of pain were asked about, the posttreatment scores increased by an average of 50% in all of the groups when compared to pretreatment. When the literature was reviewed, there were studies reporting that all 3 treatments were effective in reducing symptoms and disability. In a study conducted by Rahbar et al. [12], in which the effects of ESWT and CT on disability levels were examined, improvements were noted in the posttreatment measurements when compared to pretreatment, and the effect levels of the groups were similar in reducing disability.

All of the treatments herein had significant and positive effects on the sleep quality of the individuals. The increase in sleep quality may have been because of the decreased pain intensity and emotional stress levels. Studies conducted on MPS emphasize the importance of exercise and manipulative treatment techniques in improving sleep quality. It is reported that these methods increase relaxation by increasing the levels of endorphins and catecholamines and provide positive effects on sleep patterns [50]. The fact that the greatest effect level in the current study was in the IASTM group is in line with studies reporting the importance of manipulative treatment methods. Although the changes in sleep scores in the IASTM and ESWT groups were higher than in the CT group, this difference was not statistically significant level. When interpreting this result, the pretreatment VAS night scores must also be considered. When the contradictory answers given by the individuals in the IASTM group to both the VAS night and NOOS sleep scores before the treatment were examined, they scored significantly lower on the VAS night scores than the other groups. On the contrary, the same individuals had more severe sleep problems in the answers they gave in the sleep section of the NOOS questionnaire. This suggests the possibility that factors other than pain may have affected the sleep quality in the IASTM group. Dubrovsky et al. [51] evaluated sleep quality in individuals with MPS using polysomnographic measurements and the Pittsburgh Sleep Quality Index. Individuals were surveyed with high levels of sleep disturbance and poor sleep quality. Although they reported high-level sleep disorders and bad sleep quality in the questionnaire, the polysomnographic measurements did not provide results consistent with the feedback of the individuals. Standard physiological measures in these individuals showed insufficient evidence of sleep disturbance. These results point out that the poor sleep quality reported in MPS might be associated with other factors such as depressive symptoms and mood changes. This also needs to be taken into account when interpreting sleep reports.

The effects of the treatment modalities on the patients’ quality of life were also similar. Significant increases were detected in the quality of life scores in all 3 groups when compared to pretreatment. There are very few studies examining the quality of life in cervical MPS. In a pilot study conducted by Erden et al. [25], individuals were divided into 2 groups, as the CT group and treatment group, in which IASTM and CT were combined. The treatment was applied was successful when compared to CT only in terms of the number of days related to emotional health, and no significant differences were detected between the quality of life in the groups in terms of other subparameters. In another study, in which IASTM and HP were evaluated in terms of acute effects on quality of life, both were found to be effective on quality of life, and although the improvements were more significant in the IASTM group, this difference was not statistically significant [52]. There are also studies reporting the positive effects of ESWT and CT on quality of life. Gezginaslan et al. [13] reported that ESWT and CT are effective in increasing quality of life. Rudy et al. [53] reported that the physical disabilities caused by long-term pain in individuals with chronic pain bring anxiety about losing control over their health and life in the future, and that this had major impacts on quality of life. They also emphasized that this long-term anxiety hinders the socialization of individuals and creates long-term, difficult-to-solve effects on personality profiles and psychological states. For this reason, it would not be realistic to expect that the treatment and reduction of physical disabilities and pain would immediately be reflected on quality of life.

The HADS was used in the current study to assess anxiety and depression mood levels, as well as changes in these parameters after treatment. The anxiety and depression levels were similar at the beginning of the study. When the treatments applied to the patients were compared, it was found that the effects of the 3 treatments on reducing anxiety and depression were similar. In the study of Aktürk et al. [10], in which they compared the efficacy of 4 sessions of ESWT and 10 sessions of US, no differences were detected in the levels of anxiety and depression within and between the groups in both the evaluations immediately after the treatment and 6 weeks later, but in the study of Gezginaslan et al. [13], it was reported that ESWT was superior to CT in reducing depression levels. In the present study and that of Aktürk et al. [10], the fact that the anxiety and depression levels of the participants were low when compared to the scores received before the treatment and that they were not in the risk group may not have created a significant difference between the groups. In the study of Gezginaslan et al. [13], the participants had higher pretreatment scores, the number of ESWT sessions was higher, and the number of participants was higher than in the present study. In a pilot study conducted by Erden et al. [25], it was among the first findings that IASTM provided more significant improvements in depression levels when compared to CT. Considering all of these results, long-term follow-up studies with a larger number of participants are needed to draw definite conclusions about the effects of treatments on anxiety and depression.

Although the effectiveness of the treatments was shown with the qualitative and quantitative data obtained as a result of the treatments applied in all 3 groups in the present study, the actual success of the treatments will gain meaning when to what extent the expectations of the participants can be met is determined. The level of satisfaction was evaluated by the feedback the participants gave about the success of the treatment they received. Satisfaction levels were high in all of the groups, and the satisfaction levels in the CT+IASTM group were significantly higher than the other groups. Regarding the positive effect of CT+IASTM in all of the patients in the group, it may have created a sedative and analgesic effect in patients because the mechanoreceptive stimulation given by the stroking applications in this method was more intense than in the other treatment methods. Moreover, the contact of the therapist with the patient might have increased the patient’s sense of trust and concern, causing them to be more successful in meeting the expectations from the treatment when compared to other modalities.

The comparison of modern and traditional treatment modalities for MPS and the use of valid and reliable assessment and intervention tools are the strengths of this study. The limitation of this study was that no information was received about the menstrual cycles of the individuals during the measurements. Symptoms such as depressive mood, irritability, concentration disorders, anxiety, or mood changes occur in many women during the premenstrual period [54]. These symptoms not only affect the social functionality of individuals but also cause changes in pain perception and perspective on life [55]. It must be taken into account that the fact that these hormonal cycles were not questioned might have had an impact on the feedback given by the individuals on the questionnaires.

In conclusion, IASTM combined with CT was more effective than the other treatments in increasing ROM and PPT values and reducing activity pain in the short term. In patients with ROM limitations and low PPTs, IASTM treatment can be preferred primarily in the creation of combined treatment programs. In addition, all 3 treatment methods applied to individuals with MPS reduced symptoms, disability, anxiety, and depression, and increased sleep and quality of life. Although the effects of the treatment methods on these parameters in the short term were statistically similar, follow-up studies to see the long-term effects may be valuable in terms of contributions to the literature.

## Figures and Tables

**Figure:**
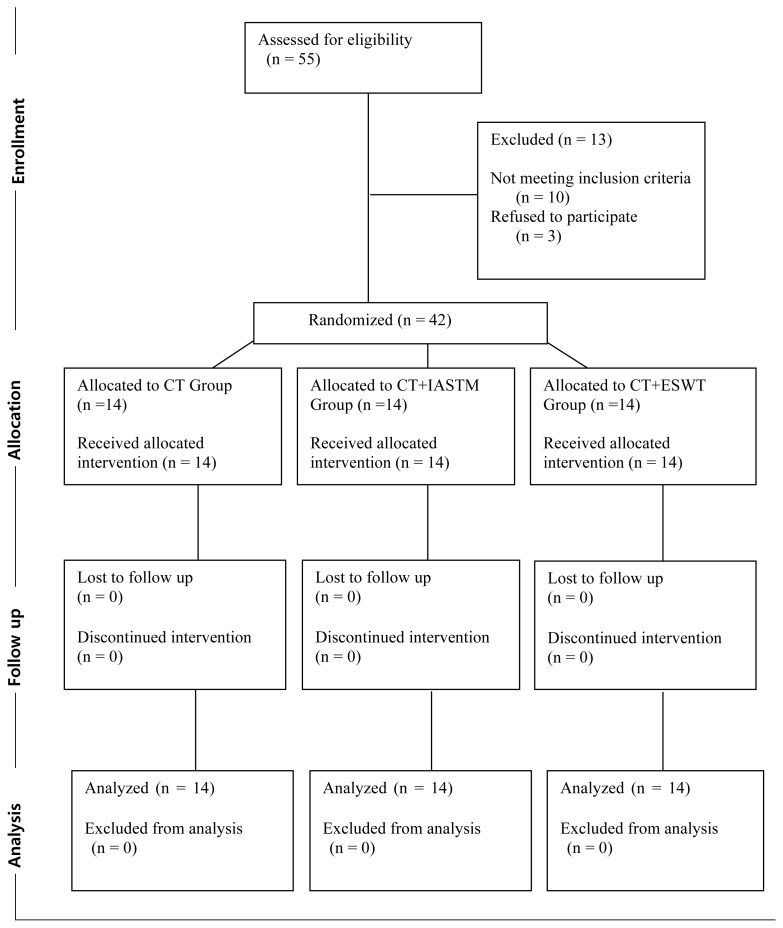
Study flow diagram.

**Table 1:** Comparison of the groups in terms of the quantitative demographic variables.

	CT (n = 14)	CT+IASTM (n = 14)	CT+ESWT (n = 14)		
	Mean ± SD	Mean ± SD	Mean ± SD	F-value	p-value
Age (year)	35.50 ± 16.32	27.71 ± 8.04	32.43 ± 13.68	1.25	0.299
Height (cm)	165 ± 6	166 ± 6	162 ± 6	1.686	0.198
Weight (kg)	65.40 ± 12.11	59.00 ± 9.82	60.43 ± 11.54	1.24	0.300
BMI (kg/m^2^)	24.80 ± 3.74	21.48 ± 3.62	23.22 ± 4.83	2.32	0.112

CT: conservative treatment, IASTM: instrument-assisted soft tissue mobilization, ESWT: extracorporeal shock wave therapy, BMI: body mass index, SD: standard deviation, F: test statistic value.

**Table 2:** Comparison of the groups in terms of the qualitative demographic variables.

		CT		CT+IASTM		CT+ESWT			
		n	%	n	%	n	%	x^2^	p-value
Marital status	Married	3	21.4	2	14.3	4	28.6	0.848	0.655
	Single	11	78.6	12	85.7	10	71.4		
Occupation	Unemployed	10	71.4	12	85.7	9	64.3	12.127	0.059
	Physically active worker	2	14.3	1	7.1	2	14.3		
	Office worker	2	14.3	1	7.1	3	21.4		
Dominant hand side	Right	13	92.9	14	100.0	13	92.9	1.050	0.592
	Left	1	7.1	0	0	1	7.1		
Presence of comorbidities	Metabolic	1	7.1	0	0	4	28.6	11.965	0.152
	Rheumatic	0	0	1	7.1	0	0		
	Neurologic	0	0	0	0	1	7.1		
	Orthopedic	1	7.1	0	0	0	0		
	No	12	85.7	13	92.9	9	64.3		
Cigarette smoker	Yes	1	7.1	1	7.1	1	7.1	-	
	No	13	92.9	13	92.9	13	92.9		
Alcohol	Yes	0	0	0	0	0	0	-	
	No	14	100.0	14	100.0	14	100.0		
Localization of the pain	Right	8	57.1	9	64.3	11	78.6	1.500	0.473
	Left	6	42.9	5	35.7	3	21.4		
Localization of the trigger point	Right upper	6	42.9	4	28.6	9	64.3	10.500	0.232
	Right center	2	14.3	4	28.6	2	14.3		
	Right lower	0	0	1	7.1	0	0		
	Left upper	6	42.9	3	21.4	3	21.4		
	Left center	0	0	0	0	0	0		
	Left lower	0	0	2	14.3	0	0		

x^2^: chi-squared statistical value.

**Table 3:** Comparison of the pretreatment scores in the treatment groups.

	CT	CT+IASTM	CT+ESWT		
	Mean ±SD	Mean ± SD	Mean ± SD	F-value	p-value
VAS rest (cm)	5.00 ± 1.24	4.14 ± 1.56	4.93 ± 2.40	1.327	0.283
VAS activity (cm)	7.00 ± 1.88	6.00 ± 2.29	7.07 ± 1.33	1.174	0.326
VAS night (cm)	5.57 ± 1.79	2.29 ± 2.81	5.57 ± 2.50	7.343	0.003*
Flexion °	40.57 ± 10.30	33.57 ± 13.36	42.93 ± 8.58	2.374	0.114
Extension °	45.07 ± 11.10	39.86 ± 17.35	42.00 ± 7.36	0.553	0.582
Right rotation °	53.29 ± 12.49	40.36 ± 14.87	56.21 ± 7.19	6.276	0.007*
Left rotation °	52.43 ± 10.29	40.71 ± 16.62	54.14 ± 9.21	3.287	0.054
Right lateral flexion °	37.71 ± 11.50	30.71 ± 7.30	35.64 ± 11.08	2.161	0.136
Left lateral flexion °	37.57 ± 9.55	32.71 ± 7.13	38.14 ± 10.03	1.830	0.181
PPT (kg/cm^2^)	4.13 ± 1.01	5.91 ± 1.69	3.35 ± 1.20	10.455	0.001*
NOOS mobility	47.70 ± 13.63	42.60 ± 18.50	41.84 ± 12.64	0.740	0.487
NOOS symptoms	43.93 ± 14.83	41.79 ± 15.01	40.00 ± 16.17	0.221	0.803
NOOS sleep disturbance	45.54 ± 15.20	42.41 ± 21.26	47.77 ± 19.86	0.232	0.794
NOOS activity and pain	33.71 ± 10.91	36.83 ± 15.61	28.80 ± 10.63	1.429	0.258
NOOS quality of life	41.96 ± 14.22	46.43 ± 21.09	41.96 ± 18.58	0.237	0.790
HADS anxiety	10.21 ± 3.51	10.29 ± 3.73	8.57 ± 4.83	0.643	0.534
HADS depression	7.07 ± 3.17	8.36 ± 3.25	7.50 ± 3.96	0.560	0.578

VAS: visual analogue scale, PPT: pressure pain threshold, NOOS: neck outcome score, HADS: Hospital Anxiety and Depression Scale, F: test statistic value,

*: p < 0.05.

**Table 4:** Within-group and between-group comparisons of the pain severity before and after treatment.

	CT^a^	CT+IASTM^b^	CT+ESWT^c^	Within-group			Between-group			post hoc
	Mean ± SD	Mean ± SD	Mean ± SD	F-value	p-value	Effect size	F-value	p-value	Effect size	
VAS rest (cm)										–
Pretreatment	5.0 ± 1.2	4.1 ± 1.6	4.9 ± 2.4	101.29	0.001	0.722	1.415	0.255	0.068	
Posttreatment	3.1 ± 1.9	1.3 ± 0.8	2.1 ± 2.6							
VAS activity (cm)										a-b (0.031) a-c (0.040) b-c (0.037)
Pretreatment	7.0 ± 1.9	6.0 ± 2.3	7.1 ± 1.3	96.93	0.001	0.713	3.071	0.043	0.125	
Posttreatment	4.6 ± 2.5	1.7 ± 1.1	3.9 ± 2.2							
VAS night (cm)										–
Pretreatment	5.6 ± 1.8	2.3 ± 2.8	5.6 ± 2.5	38.99	0.001	0.500	1.874	0.167	0.088	
Posttreatment	3.4 ± 2.6	0.4 ± 0.9	1.9 ± 2.0							

a: CT,

b: CT+IASTM,

c: CT+ESWT,

F: test statistic value, post hoc: comparison results based on the Bonferroni test.

**Table 5:** Within-group and between-group comparisons of the ROM degrees before and after treatment.

	CT^a^	CT+IASTM^b^	CT+ESWT^c^	Within-group			Between-group			post hoc
	Mean ± SD	Mean ± SD	Mean ± SD	F-value	p-value	Effect size	F-value	p-value	Effect size	
Flexion °				3268.76	0.001	0.720	2.856	0.070	0.128	–
Pretreatment	40.6 ± 10.3	33.6 ± 13.4	42.9 ± 8.6							
Posttreatment	53.1 ± 10.3	49.6 ± 9.8	51.7 ± 9.2							
Extension °										a-b (0.03) a-c (0.027) b-c (0.044)
Pretreatment	45.1 ± 11.1	39.9 ± 17.4	42.0 ± 7.4	68.68	0.001	0.638	3.576	0.037	0.155	
Posttreatment	51.3 ± 12.3	53.8 ± 14.4	56.1 ± 8.7							
Right rotation °										a-b (<0.001) b-c (0.003)
Pretreatment	53.3 ± 12.5	40.4 ± 14.9	56.2 ± 7.2	47.20	0.001	0.548	10.00	0.000	0.339	
Posttreatment	56.9 ± 12.0	56.3 ± 12.8	61.7 ± 6.8							
Left rotation°										a-b (0.015) b-c (0.044)
Pretreatment	52.4 ± 10.3	40.7 ± 16.6	54.1 ± 9.2	41.11	0.001	0.513	5.01	0.012	0.204	
Posttreatment	57.4 ± 8.4	55.6 ± 15.4	60.6 ± 6.1							
Right lateral flexion °										a-b (<0.001) a-c (0.046) b-c (0.045)
Pretreatment	37.7 ± 11.5	30.7 ± 7.3	35.6 ± 11.1	69.63	0.001	0.641	8.92	0.000	0.314	
Posttreatment	41.7 ± 11.2	46.4 ± 6.4	44.4 ± 11.5							
Left lateral flexion °										a-b (0.002) a-c (0.018) b-c (0.014)
Pretreatment	37.6 ± 9.5	32.7 ± 7.1	38.1 ± 10.0	54.27	0.001	0.582	6.95	0.003	0.263	
Posttreatment	41.1 ± 11.1	47.6 ± 6.6	47.1 ± 9.7							

a: CT,

b: CT+IASTM,

c: CT+ESWT,

F: test statistic value, post hoc: comparison results based on the Bonferroni test.

**Table 6:** Within-group and between-group comparisons of the PPT values before and after treatment

	CT^a^	CT+IASTM^b^	CT+ESWT^c^	Within-group			Between-group			post hoc
	Mean ± SD	Mean ± SD	Mean ± SD	F-value	p-value	Effect size	F-value	p-value	Effect size	
PPT (kg/cm^2^)				32.59	0.001	0.455	5.706	0.007	0.226	a-b (0.005) a-c (0.028) b-c (0.024)
Pretreatment	4.1 ± 1.0	5.91 ± 1.69	3.34 ± 1.20							
Posttreatment	4.7 ± 1.73	9.18 ± 3.58	5.14 ± 2.28							

a: CT,

b: CT+IASTM,

c: CT+ESWT,

F: test statistic value, post hoc: comparison results based on the Bonferroni test.

**Table 7:** Within-group and between-group comparisons of the NOOS and HADS scores before and after treatment.

	CT	CT+IASTM	CT+ESWT	Within-group			Between-group		
	Mean ± SD	Mean ± SD	Mean ± SD	F-value	p-value	Effect size	F-value	p-value*	Effect size
NOOS mobility									
Pretreatment	50.0 ± 11.4	42.6 ± 18.5	41.8 ± 12.6	54.65	0.001	0.584	2.97	0.063	0.132
Posttreatment	66.1 ± 18.9	75.8 ± 8.9	60.7 ± 23.9						
NOOS symptoms									
Pretreatment	43.6 ± 14.8	41.8 ± 15.0	40.0 ± 16.2	63.15	0.001	0.606	1.40	0.260	0.067
Posttreatment	62.9 ± 18.5	74.6 ± 7.20	68.2 ± 18.9						
NOOS sleep disturbance									
Pretreatment	47.3 ± 14.0	42.4 ± 21.3	47.8 ± 19.9	43.69	0.001	0.528	1.96	0.155	0.091
Posttreatment	63.4 ± 20.6	76.8 ± 12.6	72.3 ± 24.6						
NOOS activity and pain									
Pretreatment	33.3 ± 11.1	36.8 ± 15.6	28.8 ± 10.6	104.18	0.001	0.728	1.62	0.211	0.077
Posttreatment	58.6 ± 19.5	75.2 ± 14.5	66.3 ± 22.6						
NOOS quality of life									
Pretreatment	42.9 ± 12.9	46.4 ± 21.1	42.0 ± 18.6	31.03	0.001	0.443	1.12	0.338	0.054
Posttreatment	55.6 ± 20.1	71.9 ± 11.9	62.3 ± 18.9						
HADS anxiety									
Pretreatment	10.2 ± 3.5	10.3 ± 3.7	8.6 ± 4.8	23.84	0.001	0.379	1.64	0.208	0.077
Posttreatment	8.8 ± 3.6	6.6 ± 4.0	6.1 ± 3.5						
HADS depression									
Pretreatment	7.1 ± 3.2	8.4 ± 3.2	7.5 ± 4.0	24.05	0.001	0.381	1.74	0.190	0.082
Posttreatment	5.6 ± 2.8	5.1 ± 2.7	5.9 ± 3.4						

F: test statistic value, post hoc: comparison results based on the Bonferroni test,

*: corresponds to the insignificant results for post hoc.

**Table 8:** Comparison of the satisfaction levels of the groups.

	CT		CT+IASTM		CT+ESWT		x^2^	p-value
	n	%	n	%	n	%		
Unchanged	1	7.1	0	0	1	7.1	17.830	0.023
Worsening	1	7.1	0	0	1	7.1		
Mild recovered	7	50.0	3	21.4	0	0		
Significant recovered	0	0	4	28.6	4	28.6		
Completely recovered	5	35.7	7	50	8	57.1		

x^2^: chi-squared statistical value.
